# Urban Stream Burial Increases Watershed-Scale Nitrate Export

**DOI:** 10.1371/journal.pone.0132256

**Published:** 2015-07-17

**Authors:** Jake J. Beaulieu, Heather E. Golden, Christopher D. Knightes, Paul M. Mayer, Sujay S. Kaushal, Michael J. Pennino, Clay P. Arango, David A. Balz, Colleen M. Elonen, Ken M. Fritz, Brian H. Hill

**Affiliations:** 1 US EPA, Office of Research and Development, National Risk Management Research Laboratory, Cincinnati, OH, United States of America; 2 US EPA, Office of Research and Development, National Exposure Research Laboratory, Cincinnati, OH, United States of America; 3 US EPA, Office of Research and Development, National Exposure Research Laboratory, Athens, GA, United States of America; 4 US EPA, Office of Research and Development, National Risk Management Research Laboratory, Ada, OK, United States of America; 5 University of Maryland, Department of Geology and Earth Systems Interdisciplinary Center, College Park, MD, United States of America; 6 Central Washington University, Department of Biological Sciences, Ellensburg, WA, United States of America; 7 Pegasus Technical Services, Cincinnati, OH, United States of America; 8 US EPA, Office of Research and Development, National Health and Environmental Effects Research Laboratory, Duluth, MN, United States of America; NERC Centre for Ecology & Hydrology, UNITED KINGDOM

## Abstract

Nitrogen (N) uptake in streams is an important ecosystem service that reduces nutrient loading to downstream ecosystems. Here we synthesize studies that investigated the effects of urban stream burial on N-uptake in two metropolitan areas and use simulation modeling to scale our measurements to the broader watershed scale. We report that nitrate travels on average 18 times farther downstream in buried than in open streams before being removed from the water column, indicating that burial substantially reduces N uptake in streams. Simulation modeling suggests that as burial expands throughout a river network, N uptake rates increase in the remaining open reaches which somewhat offsets reduced N uptake in buried reaches. This is particularly true at low levels of stream burial. At higher levels of stream burial, however, open reaches become rare and cumulative N uptake across all open reaches in the watershed rapidly declines. As a result, watershed-scale N export increases slowly at low levels of stream burial, after which increases in export become more pronounced. Stream burial in the lower, more urbanized portions of the watershed had a greater effect on N export than an equivalent amount of stream burial in the upper watershed. We suggest that stream daylighting (i.e., uncovering buried streams) can increase watershed-scale N retention.

## Introduction

River networks provide critical ecosystem services including clean drinking water, habitat for aquatic life, and nutrient uptake. However, many streams in urban and agricultural areas have been confined in pipes and buried beneath fields, buildings, parking lots, and other elements of the human-dominated landscape. While stream burial dates to at least the Roman Empire [1], the extent of stream burial in contemporary urban centers is exceedingly high. For example, an estimated 66% of streams in Baltimore, Maryland, USA have been buried, and >98% of streams in the most densely populated areas of the city are buried [2]. Similarly, stream burial has reduced the drainage density in urbanized watersheds in Maryland and North Carolina (USA) by 58% and 40%, respectively [3,4]. Stream burial is not unique to North America, but has also been reported in Asia [5] and several countries in Europe [6] where 20% and 15% of all streams in Switzerland and Denmark are buried, respectively [7,8]. Given the expected expansion of urban areas worldwide [9], stream burial will likely increase over the coming decades. Although stream burial can comprise a significant alteration to headwater ecosystems [10,11], little is known about its environmental impacts on water quality and the ecosystem services provisioned by streams and rivers [12].

An important ecosystem service that may be affected by urban stream burial is nitrate (NO_3_^-^) uptake. Humans have doubled the rate of nitrogen input into the terrestrial nitrogen (N) cycle, causing more NO_3_^-^ to enter streams and rivers [13] resulting in the eutrophication of downstream estuaries and coastal waters [14]. However, streams can reduce N loading to downstream ecosystems by removing NO_3_^-^ from the water column via biological uptake [15,16]. Nitrate uptake in streams occurs primarily through denitrification and assimilation. Denitrification is an anaerobic microbial process where NO_3_^-^ is converted to dinitrogen (N_2_) or nitrous oxide (N_2_O) gas and emitted to the atmosphere, resulting in a permanent removal of NO_3_^-^ from the stream. Assimilation occurs when NO_3_^-^ is incorporated into biomass, which can be buried in sediments and stored for long periods of time or released back to the environment in the form of ammonium (NH_4_^+^). The NH_4_^+^ can then be converted back to NO_3_^-^ via microbial nitrification. Therefore, only a fraction of NO_3_^-^ uptake that occurs in streams results in a permanent removal of N from the ecosystem. Nevertheless, biological N removal in streams and rivers can be substantial. For example, biological activity removes approximately 50% of the N delivered to the Mississippi River drainage network, reducing downstream transport to the N-limited Gulf of Mexico [17]. Although human activities such as channelization, development in the riparian zone, and the establishment of impervious surfaces can reduce the N-removal capacity of urban streams [18], many urban streams exhibit some level of resiliency to these stressors and function as important sites of N removal in urban watersheds [16,19]. This may not be the case for buried streams, however, where the complete loss of light and photosynthesis, elimination of direct organic matter inputs from streamside vegetation, and a greatly simplified stream channel represent extreme disturbances that may overwhelm the resiliency of the stream channel and result in greatly reduced NO_3_^-^ uptake rates in large portions of urban stream networks.

Until recently, the effect of stream burial on NO_3_^-^ uptake was unknown, which motivated studies that used whole-stream ^15^NO_3_^-^ isotope tracer additions to measure NO_3_^-^ uptake seasonally in paired buried and open stream reaches of three streams in Cincinnati, Ohio [20] and three streams in Baltimore, Maryland (USA) [21]. Both studies showed that the absence of light and low carbon availability reduced NO_3_^-^ uptake in all buried streams. While these studies are among the first to document the effects of stream burial on NO_3_^-^ uptake in urban centers, they were conducted at the reach scale and little is known about how the effects may propagate to the watershed scale. This is an important consideration because the cumulative effect of reduced NO_3_^-^ uptake in many short reaches distributed throughout a watershed could be considerable at the watershed scale.

Here we present work aimed at two key objectives: (1) To synthesize the results of the Baltimore and Cincinnati studies to compare the NO_3_^-^ uptake response to stream burial between two distinct urban areas and generate NO_3_^-^ uptake data for our second objective, which is (2) To explore the effect of urban stream burial on watershed-scale NO_3_^-^ export under different extents (e.g., 0–100%) and spatial distributions of urban stream burial in a river network using a spatially-explicit mechanistic model. Mechanistic models allow us to investigate hypothetical scenarios and to scale measurements from the stream-reach to the watershed, which is the scale at which management practices are evaluated.

## Materials and Methods

### Syntheses of Field Measurements

**Fig 1:**
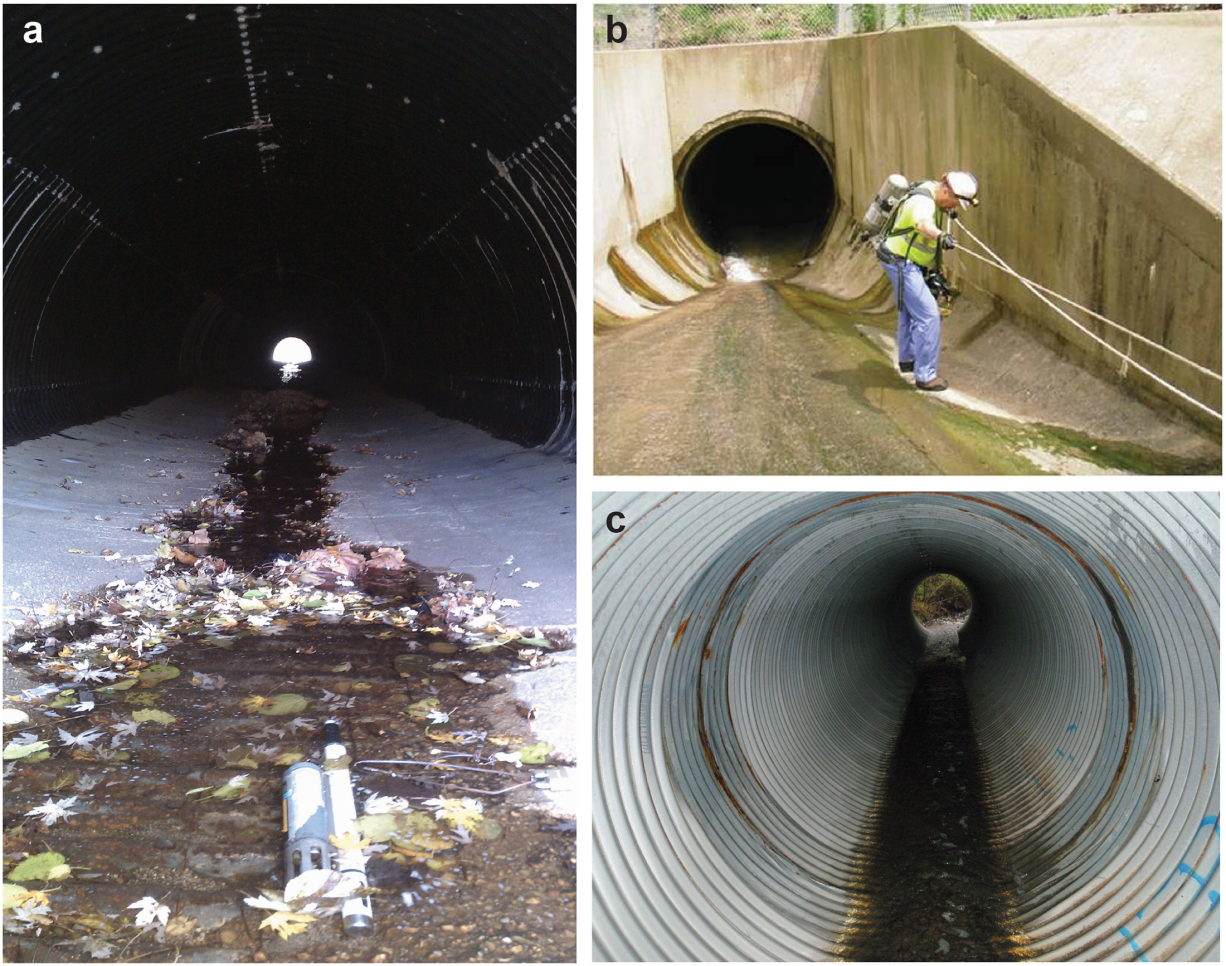
Stream burial is an extreme, but ubiquitous, consequence of urbanization in stream ecosystems. The buried stream channels in the cited studies were constructed from various materials including (a) a cement-lined corrugated metal pipe in Baltimore, Maryland (USA), (b) a concrete tunnel in Cincinnati, Ohio (USA), and (c) a corrugated metal pipe in Cincinnati.

We synthesize field measurements of NO_3_^-^ uptake in buried and open reaches in the cities of Cincinnati, Ohio and Baltimore, Maryland, USA that were presented separately in Beaulieu et al. [20] and Pennino et al. [21]. The studies entailed detailed measurements of NO_3_^-^ uptake rates in the buried and open reaches of three streams in each city across four seasons. The six study streams were small (discharge: 0.5–61 L s^-1^; median 5.2 L s^-1^; Fig 1), drained urbanized basins (16–48% impervious cover), and had moderately high NO_3_^-^ concentrations (164–2529 μg N L^-1^; median: 986 μg N L^-1^;Table 1). The individual shown in Fig 1B has given written informed consent (as outlined in PLOS consent form) to publish the image. The streams in Baltimore are part of the Baltimore Ecosystem Study of the Long Term Ecological Research (LTER) network and flowed beneath roadways. The streams in Cincinnati flowed beneath roadways, parking lots, and buildings. Nitrate uptake was measured using whole-stream ^15^NO_3_^-^ isotope tracer additions (see original publications for details). Nutrient spiraling theory [22] was used to calculate (1) the first order NO_3_^-^ uptake rate constant (*k*, in units of per minute), (2) the NO_3_^-^ uptake length (*S*_*w*_), which is the average distance a NO_3_^-^ molecule travels downstream before being removed from the water column, and (3) the NO_3_^-^ uptake velocity (*ν*_*f*_), a metric that accounts for the influence of water depth and velocity on *S*_*w*_ and is an index for biological NO_3_^-^ demand. Nitrate uptake in the river network model (see River Network Model and Site Description below) was specified with *k*, while the statistical analysis of the field measurements focused on *ν*_*f*_ and *S*_*w*_. To quantify the degree to which stream burial affects NO_3_^-^ uptake, we calculated the ratio of *ν*_*f*_ and *S*_*w*_ in the open reach to that of the buried reach for each stream. We used paired t-tests to determine if log-transformed NO_3_^-^ uptake indices (*S*_*w*_ and *ν*_*f*_) differed between buried and open reaches and t-tests to determine if the magnitude of the burial effect differed between the two cities. We used a general linear model (glm) to relate *ν*_*f*_ to stream NO_3_^-^ concentration. The glm was fit with a variance structure which allows for the range of model residuals to differ for each stream by reach combination in the data set. The alternative variance structure was needed to assure the model met the assumptions of parametric statistics (i.e., normality, homogeneity, and independence of residuals).

**Table 1:** Mean (range) nitrate (NO_3_^-^) concentration, discharge (Q), NO_3_^-^ uptake velocity (ν_f_), NO_3_^-^ uptake length (S_w_), and the first order NO_3_^-^ uptake rate constant (*k*) measured during four seasons from buried and open reaches in three streams in Cincinnati, Ohio (USA) and three streams in Baltimore, Maryland (USA). Data originally reported in Beaulieu et al. [20] and Pennino et al. [21].

City	Reach	NO_3_^-^ (μg N L^-1^)	Q (L s^-1^)	*ν*_f_ (mm min^-1^)	S_w_ (m)	k (min^-1^)
Baltimore	buried	1427 (447–2511)	5.9 (0.6–14.7)	0.06 (0.007–0.28)	3975 (421–8747)	0.0029 (0.0157–0.0003)
	open	1566 (390–2529)	4.9 (0.5–13.9)	0.25 (0.083–0.62)	1104 (83–2921)	0.0036 (0.0114–0.0005)
Cincinnati	buried	491 (226–811)	13.2 (0.9–52.8)	0.16 (0.003–0.74)	14491 (707–74131)	0.0037 (0.02–0.0001)
	open	461 (164–822)	14.1 (1.7–60.5)	0.50 (0.053–1.98)	1835 (89–8014)	0.0048 (0.0119–0.0003)

### River Network Model and Site Description

**Fig 2:**
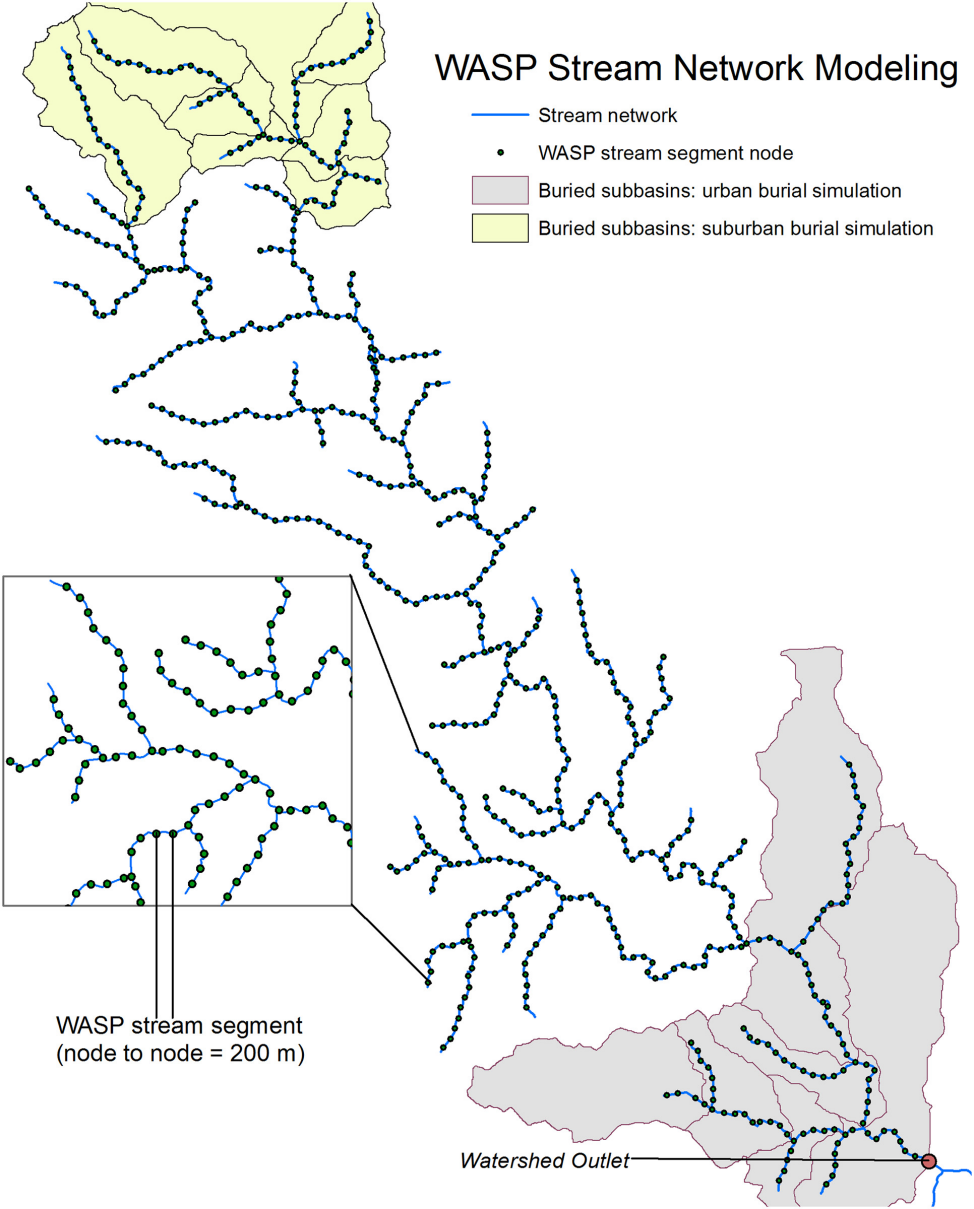
The stream network used for WASP7 modeling. The nodes defining the upstream and downstream extent of each of the 675 model stream segments are represented by green dots. Stream segments in the yellow and grey shaded sub-basins reflect the suburban and urban burial scenarios, respectively.

The objective of the river network modeling was to simulate and assess the relative changes in watershed-scale NO_3_^-^ export across a gradient of urban stream burial scenarios. Model simulations were conducted in the Gwynns Falls Watershed (171 km^2^), Maryland, USA, a subwatershed that discharges to the Chesapeake Bay. The three streams included in the Pennino et al. [21] investigation of NO_3_^-^ uptake in buried streams (study described above) are located within this watershed, a system with predominately urban land cover/land use. While a small portion of the upper watershed is comprised of suburban land cover with interspersed urban parks and preserves, the lower watershed and its outlet are located within the City of Baltimore, Maryland. Elevations range from approximately 183 m in the headwaters to 0 m at the outlet. Our river network modeling for the Gwynns Falls Watershed was conducted in two phases: (1) delineation and characterization of the stream network and (2) model simulations. The stream network was developed by merging the watershed’s 1:24000 National Hydrography Dataset (http://nhd.usgs.gov/index.html) stream lines with manually digitized storm drains from a spatial data layer provided by Baltimore County, Maryland (S1 Fig). This network was then segmented uniformly into 675 reaches (length 200 m) for model simulations (Fig 2). We used the Water Quality Analysis and Simulation Program (WASP, version 7.52; herein referred to as WASP7) to simulate and assess the relative cumulative effects of stream burial on watershed NO_3_^-^ export at the outlet of the Gwynns Falls Watershed, located at the US Geological Survey Washington Boulevard stream gage (USGS 01589352), under different burial scenarios. Because the Gwynns Falls stream network contained more stream origins and confluences than WASP7 could accommodate (maximum = 50), we removed all streams whose length was less than two modeling reaches (400 m) long. This resulted in the removal of less than 20% of the stream network and the model system should be considered an idealized river network suitable for our objective of assessing the relative cumulative effects of stream burial at the outlet of a mesoscale watershed.

The WASP7 model is a flexible, mechanistic, spatially and temporally resolved dynamic mass balance model, which simulates volumetric stream flow, velocity, and concentrations of environmental constituents in each stream segment (Fig 2). WASP7 incorporates the biological and hydrological processes that determine NO_3_^-^ uptake within a spatially resolved framework to simulate NO_3_^-^ fate and transport in rivers. The NO_3_^-^ uptake rate constants (*k*) for open reaches in the model were specified as the mean summer-time NO_3_^-^ *k* measured in the open reaches in Cincinnati and Baltimore (0.00543 min^-1^). The NO_3_^-^ uptake rate constant for buried reaches (0.00121 min^-1^) was set at 4.5 times lower than the open reaches, based on the mean ratio of open to buried *k* values measured in each stream during the studies (Table 1). We chose to average the results from the two cities because the effect of burial on NO_3_^-^ uptake did not differ between the two cities (see Syntheses of Field Measurements in Results and Discussion). We modeled NO_3_^-^ uptake using first-order reaction kinetics, which is consistent with the field measurements at our study sites and previous river network modeling efforts [23,24]. Thus, NO_3_^-^ concentration is a function of the uptake rate constant and water residence time (i.e., [NO_3_^-^]_*t*_ = [NO_3_^-^]_0_*e^-kt^, where *k* is the rate constant and *t* is the water residence time).

We specifically focused on the summer baseflow period to isolate the watershed-scale effects of stream burial from highly variable hydrological conditions during other portions of the year. Manning’s roughness coefficients for the buried and open reaches were specified as 0.005 and 0.04, respectively, based on the mean ratio of buried to open reach water velocities measured in the studies. This resulted in water velocities that were 3.6 times greater in the buried than in the open reaches. We used the WASP7 model to simulate NO_3_^-^ stream concentrations at every stream segment throughout the model watershed, and used the output NO_3_^-^ concentration and outflow at the watershed’s outlet to calculate NO_3_^-^ export.

Our model simulations reflect hypothetical patterns of stream burial in the watershed. In the first set of simulations, we varied the degree of stream burial at 5% intervals from 0 to 100% by evenly distributing buried reaches throughout the system. For example, every tenth and every second stream segment was buried under the 10% and 50% burial scenarios, respectively. In the second set of scenarios, we ran simulations where burial affected either water velocity or NO_3_^-^ uptake, but not both. These simulations were conducted to evaluate the relative importance of either factor. Finally, to explore the relative importance of urban and suburban stream burial on watershed-scale NO_3_^-^ export we ran simulation scenarios with no stream burial in the watershed, complete burial of streams in suburban areas, and complete burial of streams in urban areas (Fig 2). Additional details on WASP7 simulation modeling including a detailed stream network, a watershed figure, hydraulic input parameters, and additional information on selected patterns of stream burial can be found in the Supporting Information (S1 Fig and S1 Table).

## Results and Discussion

### Syntheses of field measurements

Nitrate uptake length (*S*_*w*_), the average downstream distance traveled by NO_3_^-^ molecules before being removed from the water column, was 18 (SE = 11, p<0.001) times longer in buried than open streams in Cincinnati and Baltimore (Table 1). Stream burial also decreased the NO_3_^-^ uptake velocity (*ν*_*f*_, mm min^-1^), a metric that accounts for the influence of water depth and velocity on NO_3_^-^ uptake length and is an index of biological NO_3_^-^ demand, in both cities, indicating that stream burial substantially impairs NO_3_^-^ uptake in streams (p<0.001). The magnitude of the effects were variable, but did not differ between cities (p≥0.35), suggesting that site specific factors, such as pipe construction material or buried stream length, were more important determinants of NO_3_^-^ processing rates in buried streams than geographic region. Mulholland et al. [16] reported that NO_3_^-^ *ν*_*f*_ was negatively related to stream NO_3_^-^ concentration, reflecting a decline in NO_3_^-^ uptake efficiency at elevated NO_3_^-^ concentrations, but our analysis of the field measurements found only weak support for this relationship (p = 0.06), possibly because NO_3_^-^ concentration was relatively high at all study sites (range: 164–2529 μg N L^-1^; Table 1).

**Fig 3:**
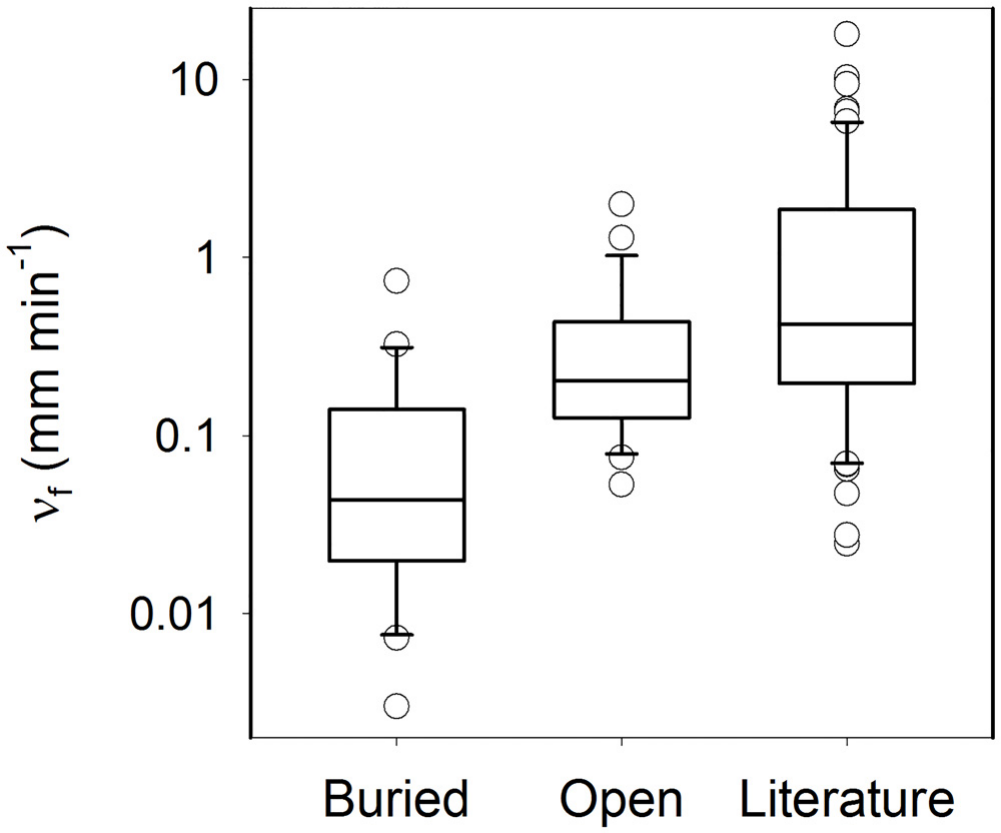
Box and whisker plots of nitrate uptake velocity (ʋ_f_) in the buried and open reaches in Cincinnati, Ohio and Baltimore, Maryland, as reported in Beaulieu et al. [20] and Pennino et al. [21]. Literature data were derived from a recent survey of 72 streams spanning several biomes and land-use conditions [16]. Plots display 10^th^, 25^th^, 50^th^, 75^th^, and 90^th^ percentiles and individual data points outside the 10^th^ and 90^th^ percentiles. Nitrate uptake velocity was 13 times greater in open than buried reaches (p<0.001, paired *t*-test).

Compared to a recent survey of 72 streams [16], both the open and buried study sites have low NO_3_^-^ *ν*_*f*_ values (Fig 3), which likely reflects the poor ecological condition of these urban streams. This was particularly true for the buried reaches which had among the lowest *ν*_*f*_ values ever reported, although the values spanned two orders of magnitude. The large range of NO_3_^-^ *ν*_*f*_ values observed in the buried streams indicate that the effect of burial on NO_3_^-^ uptake is not uniform, but varies among streams. Identification of the factors that determine the extent to which burial affects NO_3_^-^ uptake could result in management strategies to enhance the NO_3_^-^ removal capacity of currently buried streams and mitigate the loss of uptake capacity in future stream burial projects.

### River network model

**Fig 4:**
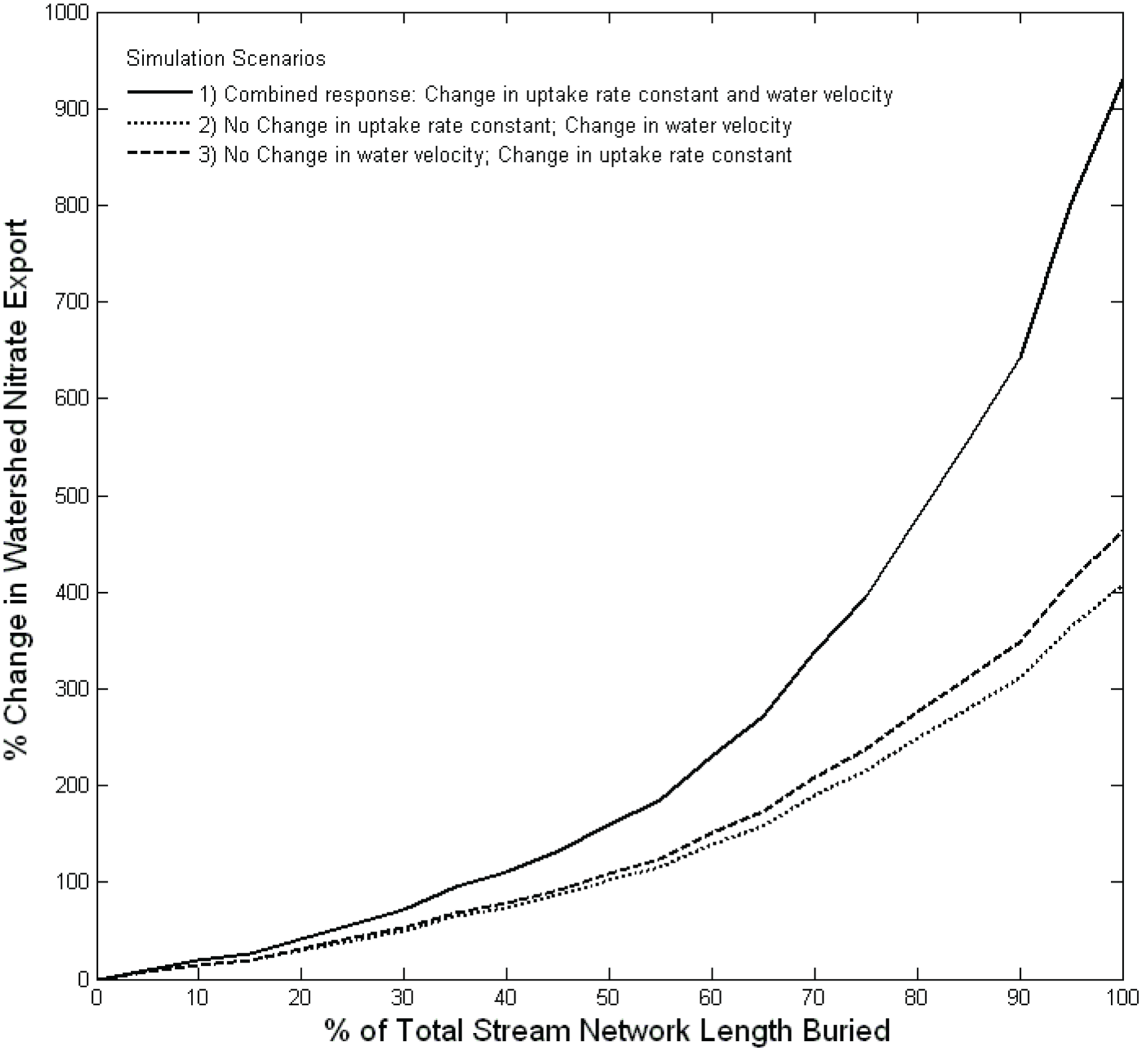
Percent change in nitrate export in response to stream burial simulation scenarios. The simulation scenarios involve an even distribution of burial across the watershed with incremental increases of 5% and include: 1) Allowing both uptake rate constants and water velocities to change in response to burial (Combined response); 2) Allowing water velocity to change following burial, but holding uptake rate constants at open reach values; and 3) Allowing uptake rate constants to change following burial, but holding water velocities at open reach values.

**Fig 5:**
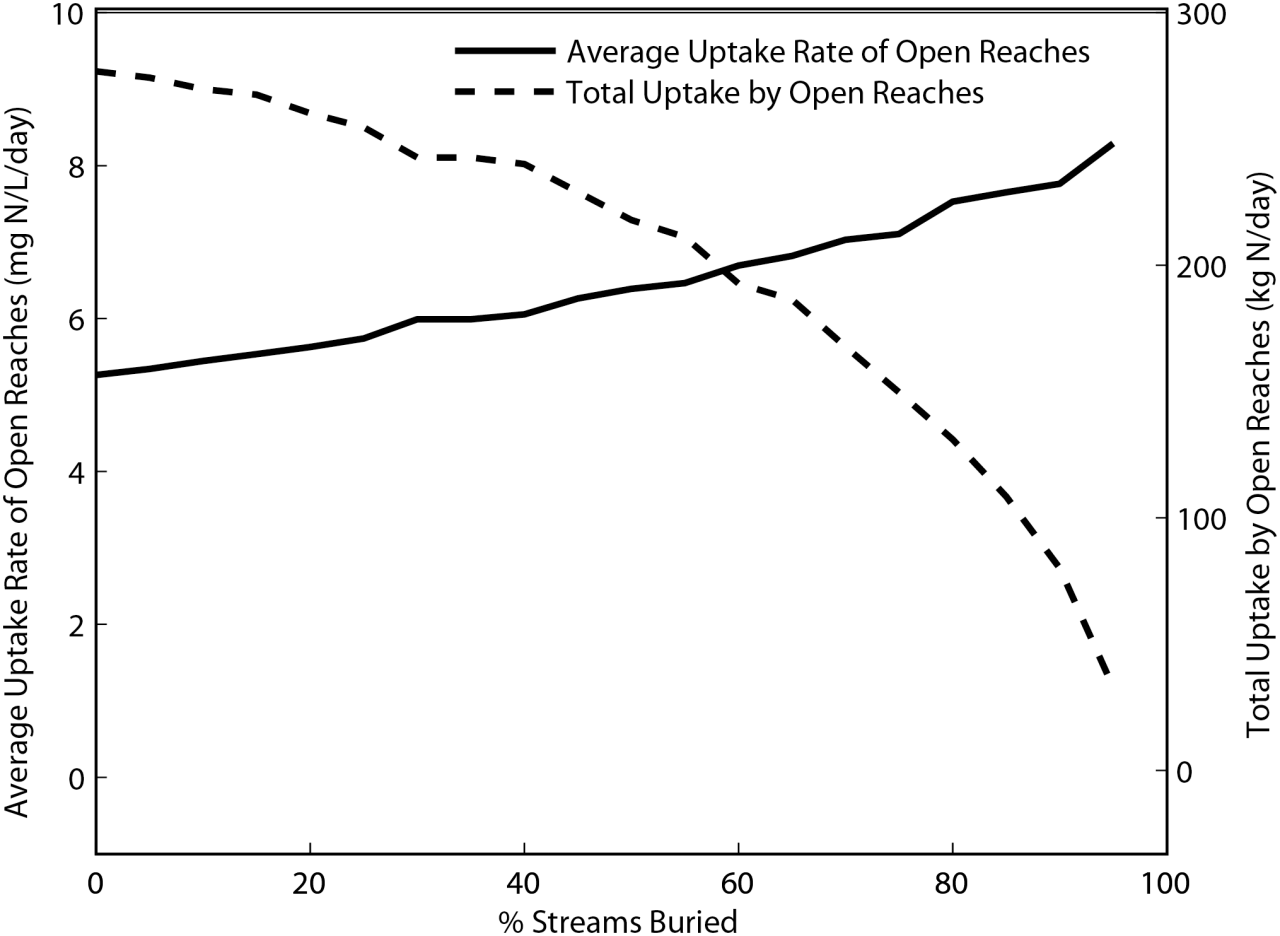
Results of simulation scenarios involving an even distribution of burial across the watershed with incremental increases of 5%. The primary y-axis and solid line represent the average volumetric NO_3_^-^ uptake rate among in the open reaches. The secondary y-axis and dashed line represent total NO_3_^-^ uptake in the open reaches.

In the first set of the river network model simulations, in which we varied the degree of stream burial at 5% intervals from 0 to 100%, results suggest that increasing stream burial would cause a gradual increase in watershed NO_3_^-^ export up to approximately 50% burial, after which increases in export become more pronounced (Fig 4, solid black line). The non-linear relationship between burial extent and watershed NO_3_^-^ export results from changes in NO_3_^-^ uptake rates in both buried and open reaches as burial extends through the river network. Uptake rates are reduced in buried reaches, resulting in higher NO_3_^-^ concentrations in downstream open reaches, which stimulates NO_3_^-^ uptake rates (Fig 5, solid line). Elevated uptake rates in open reaches compensates, in part, for the loss of NO_3_^-^ uptake in buried reaches, particularly when only a small fraction of the stream network is buried and open reaches are relatively abundant. As burial becomes more widespread, however, open reaches become increasingly rare and cumulative NO_3_^-^ uptake in the open reaches declines rapidly (Fig 5 dashed line), despite high NO_3_^-^ uptake rates. Thus, at the early stages of urban development, NO_3_^-^ export is relatively insensitive to stream burial, partly because open reaches compensate for reduced uptake in the buried reaches. As burial becomes more pervasive, however, NO_3_^-^ removal in the few remaining open reaches becomes overwhelmed and small increases in stream burial result in progressively larger increases in NO_3_^-^ export, especially after >50% of streams in the watershed have been buried. Moreover, because we modeled NO_3_^-^ uptake using first-order reaction kinetics, changes in these parameters following stream burial will produce a non-linear change in NO_3_^-^ concentration. This, in turn, results in a non-linear relationship between NO_3_^-^ export and extent of stream burial (Fig 4).

Stream burial can affect NO_3_^-^ export by changing biological NO_3_^-^ uptake or hydrologic conditions. Thus, our second set of scenarios assisted in determining the relative importance of these factors. Both simulations yielded a non-linear increase in NO_3_^-^ export with increasing burial (Fig 4). The shape of the curves were similar to that observed when water velocity and NO_3_^-^ uptake were allowed to vary simultaneously (Fig 4, solid black line), though the magnitude of each individual effect was approximately half that of the combined effect. While the magnitude of the hydrological and biological effects were similar in the simulation modeling, their relative importance is likely to vary across different systems. For example, Beaulieu et al [20] reported that the hydraulic effect was greatest when burial narrowed the channel and increased water velocity.

The location of stream burial in the river network may also be an important factor regulating changes in NO_3_^-^ export. Burial in our study system is predominant within the urban core (along the river mainstem) and suburban areas (largely headwater reaches) [2]. Therefore, our third set of simulation scenarios examined the effects of no stream burial in the watershed, complete burial of streams in suburban areas, and complete burial of streams in urban areas (Fig 2). The cumulative buried stream length used in the urban and suburban scenarios was 1700 m, which is the total stream length in the suburban areas and is equivalent to 12.2% of the total stream length in the watershed. Our simulation results suggest that NO_3_^-^ export would increase by 79% and 1% following burial in the urban core and suburbs, respectively, relative to zero burial conditions. This suggests that stream burial in the suburbs, which primarily occurs along headwater streams, would potentially have less impact on NO_3_^-^ export compared to the same extent of burial in the urban core of the watershed, which primarily affects the river mainstem.

The results of the suburban and urban development simulations were unexpected because several studies [15,25] have emphasized the importance of headwater streams in watershed nutrient retention, due to their large benthic surface area relative to the overlying water volume, which leads to greater contact and exchange of water and nitrogen with stream sediments. However, the 1700 m of streams buried in the suburban development scenario comprised a lower total volume of in-stream water storage compared to the 1700 m of higher order, largely mainstem reaches buried in the urban development scenario. The higher total volume of in-stream water storage and long residence times in the lower urban-watershed affords greater potential for uptake in the urban reaches, and thereby a greater influence of urban burial on downstream NO_3_^-^ export in the model watershed. Further, the urban stream reaches drained directly to the watershed outlet, whereas the suburban stream reaches were located in the upper portions of the watershed. Therefore, the loss of uptake in the buried suburban reaches was compensated, in part, by increased NO_3_^-^ uptake in the intervening open reaches as NO_3_^-^ was mobilized downstream (Fig 2).

## Summary and Implications

The results of our simulation modeling indicate that the effect of stream burial on watershed-scale NO_3_^-^ export is nonlinear and variable depending on the length, watershed extent, and spatial arrangement of buried stream segments. These findings have important implications for N management in urbanized watersheds (e.g., managing for water quality under the European Union Water Framework Directive [26]), and we suggest future work should focus on identifying management actions that can restore NO_3_^-^ uptake and removal to buried streams. For example, our data strongly suggest that uncovering buried streams via ‘daylighting’ may increase in-stream NO_3_^-^ uptake, leading to reduced NO_3_^-^ export to downstream ecosystems. Although there are many examples of daylighted streams around the world [6,27,28], the effect on NO_3_^-^ uptake has not been documented, and further research should be conducted to identify effects under different land-use, nutrient load, and climate conditions. There may also be management actions short of daylighting that can enhance NO_3_^-^ uptake and removal in buried streams. For example, a manageable feature of buried streams that may influence NO_3_^-^ uptake and removal is pipe width. Wider pipes, and therefore wider buried stream channels, lead to shallower streams with enhanced interaction between water-column nutrients and biologically active stream sediments that can lead to improved NO_3_^-^ uptake [20,29]. Whether the pipe has an open bottom, allowing some degree of groundwater-surface water exchange, or is completely encased in impervious material, may also influence NO_3_^-^ uptake. Several studies have found that the degree of temporary surface-water storage in stream sediments (i.e., transient storage) enhances NO_3_^-^ uptake [18,30], therefore open-bottomed structures may support greater uptake than structures that completely encasethe stream.

This work is among the first to explore the effects of stream burial on NO_3_^-^ export at the watershed scale and it raises important questions for additional research. For example, our model simulates NO_3_^-^ fate and transport during the summer baseflow period to isolate the effects of urban stream burial, but we do not address how temporal dynamics, such as storm flows and variations in hydrological conditions, may influence the role of stream burial in exacerbating watershed NO_3_^-^ export [31]. The importance of stream burial on NO_3_^-^ export from urban watersheds is also likely to vary across large geographic scales. For example, urban development in the Northeastern United States often results in the loss of streams due to burial [2], whereas cities in arid environments often have a higher density of surface water than the surrounding environment due to extensive canal building [32]. Moreover, our model required removal of a limited number of headwater streams for efficient computation; their inclusion in a less complex river network model could provide new insights. Incorporating these temporal and geographic patterns into the simulation modeling therefore represents an important research need. Furthermore, future research that incorporates uncertainty in field measurements and the variability of individual reach characteristics, such as culvert types, channel dimensions, and stream gradients, which all may influence channel velocity, residence time, and uptake rates, into watershed-simulations may provide additional useful insights. Expanding work to focus on the effects of stream burial on nitrate uptake and watershed export could also focus on agricultural areas where nutrient sources are high and stream burial is known to occur at elevated rates [33,34]. Finally, the WASP7 model simulates NO_3_^-^ uptake as a permanent removal process; however, the model was parameterized with measurements of NO_3_^-^ uptake that included both temporary and permanent removal process (i.e., assimilation and denitrification, respectively). Therefore, the model results represent an upper bound to the amount of NO_3_^-^ removal that would be lost from the river network following stream burial. Field based measurements of the relative importance of assimilation and denitrification in open and buried streams would allow for more constrained estimates of the effect of stream burial on watershed NO_3_^-^ export.

Overall, we suggest that stream burial should be recognized as an important disturbance to urban biogeochemical cycles and should be considered in watershed N management plans. Field measurements and simulation modeling indicate that management actions designed to protect streams from burial and which encourage ‘daylighting’ of buried streams may lead to improved water quality in urban ecosystems. This is particularly important in systems similar to Gwynns Falls Watershed, where the management of N loading to estuaries that drain developed or mixed land-use basins, such as Chesapeake Bay, is critical.

## Supporting Information

S1 Fig Stream network and watershed used for WASP7 modeling. (DOCX)

S1 Table WASP7 hydraulic input parameters. (DOCX)
