# Supplementary material for: Urban Stream Burial Increases Watershed-Scale Nitrate Export
Source: PLoS One. 2015 Jul 17;10(7):e0132256. doi: 10.1371/journal.pone.0132256 (PMC4505844; doi:10.1371/journal.pone.0132256)
Supplement: S1 Fig — (DOCX) [file pone.0132256.s001.docx]

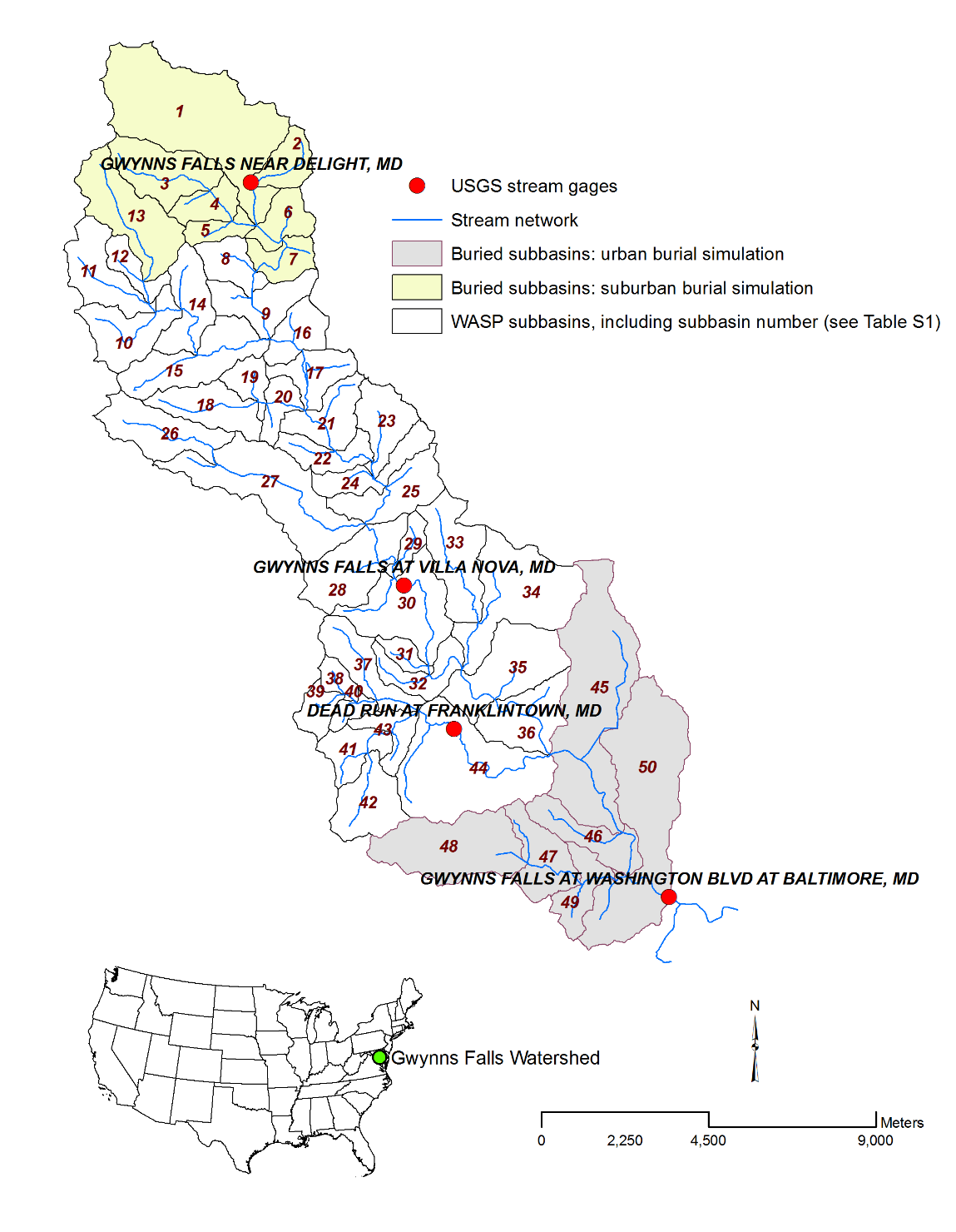


Fig S1. The Gwynns Falls Watershed and the stream network used for WASP modeling. Changes in NO_3_^-^ export were assessed at the Gwynns Falls at the Washington Boulevard stream gage (USGS 01589352). Stream segments in the yellow and grey shaded sub-basins reflect the suburban and urban burial scenarios, respectively. Sub-basins used for WASP modeling are numbered and can be cross-reference with stream segment information in TS02. Average summer baseflow NO_3_^-^ concentrations and discharge were used as inputs for each of the 50 sub-basins of the WASP model.
