# Supplementary material for: Urban Stream Burial Increases Watershed-Scale Nitrate Export
Source: PLoS One. 2015 Jul 17;10(7):e0132256. doi: 10.1371/journal.pone.0132256 (PMC4505844; doi:10.1371/journal.pone.0132256)
Supplement: S1 Table — (DOCX) [file pone.0132256.s002.docx]

**Supplemental Tables.**

Table S1. WASP hydraulic input parameters for each simulated stream segment under estimated current burial conditions.

1. WASP modeling methods

2.1 Stream Network

As a first step in the scenario simulations, we delineated the watershed and stream segments as input data to WASP. We used a 1:24000 NHD stream network as the base data layer. We manually digitized storm drains greater than or equal to 0.8 m wide (equal to or greater than the approximate median pipe size in the watershed) and 200 m long (the length of WASP modeling segments), based on the Baltimore County Storm Drain layer issued from Baltimore County, Maryland, Office of Information Technology. We merged the storm drain layer with the NHD layer to create a modeling stream network for Gwynns Falls Watershed (FS03). We subdivided the stream network into 200m long WASP modeling segments to create a uniform network for the watershed-scale simulations. This length falls near the median of the stream reaches measured in the study and resulted in 675 stream modeling segments. We identified buried streams as the digitized storm drain segments in addition to the NHD stream network segments identified as artificial paths or path connectors. Because WASP can receive a maximum of 50 streamflow inputs as boundary conditions across a river network, simplification of the network in this large watershed was required. This resulted in the removal of several low order streams throughout the watershed, which may result in conservative estimates of the influence of low order streams on downstream NO_3_^-^ export.

2.2 WASP Model

Our goal was to conduct watershed-scale WASP simulations as an exploratory modeling exercise to estimate the relative change in watershed NO_3_^-^ export in response to various stream burial scenarios. Our approach used a simplified representation of the Gwynns Falls Watershed combined with model parameter values that were directly measured, estimated, or extracted from the literature. Therefore, we did not aim to conduct rigorous model calibration; we developed a model based on estimated current conditions from which we could assess relative changes to nitrate under different levels of urbanization and stream burial in the watershed.

WASP is a flexible dynamic, differential mass-balance modeling framework used to simulate the fate and transport of a variety of contaminants in surface water systems. WASP been applied for a variety of research and regulatory questions [[*Knightes et al.*, 2009](#_ENREF_8); [*Lin et al.*, 2011](#_ENREF_9)] and has recently been integrated into a modeling system for environmental assessments [[*Johnston et al.*, 2011](#_ENREF_7)]. Here, we use WASP7 (referred to as WASP), an enhancement to the original WASP model [[*R.B. Ambrose Jr*, 1987](#_ENREF_1); [*Robert B. Ambrose Jr*, 1988](#_ENREF_2)], without additional modifications to the code. The WASP model simulates water volume and velocity in each segment using the kinematic wave option, and simulates NO_3_^-^ concentration in each segment. Detailed equations for the WASP model can be found elsewhere [[*R.B. Ambrose Jr*, 1987](#_ENREF_1); [*Robert B. Ambrose Jr*, 1988](#_ENREF_2)].

We simulated in-stream NO_3_^-^ uptake as a first order decay rate (k; min^-1^, see equation 1) estimated from measured data at both the Cincinnati and Baltimore sites. We applied one uptake rate to buried streams (0.00121 min^-1^) and one to open streams (0.00543 min^-1^). Based upon measured conditions during the 2011 sampling season, velocity (m d^-1^) in buried streams was approximately 3.5 times greater than that in open channels. We adjusted stream modeling segment velocities in the WASP model to approximate this trend by modifying the Manning’s roughness coefficient. Based upon these simulations, we applied a final roughness coefficient for buried (Manning’s = 0.005) and open (Manning’s = 0.04) streams.

Because the WASP model structure allows a maximum of 50 stream flows as boundary conditions for river systems, we divided the watershed into 50 sub-basins (FS02), each receiving daily streamflow and NO_3_^-^ concentration inputs. We input uniform average daily summer base flows (m^3^ s^-1^) estimated for the 21 June through 22 September 2011 period, the year that summer uptake rates were measured, based on the records from the closest downstream USGS stream gage to each sub-basin (FS03). We calculated average percent baseflow from the streamflow records using the 2006 version of Baseflow Program [[*Arnold and Allen*, 1999](#_ENREF_3); [*Arnold et al.*, 1995](#_ENREF_4)] and scaled the results using an area to discharge ratio for each sub-basin. We estimated a single average summer NO_3_^-^ concentration from three of the four USGS station records. We averaged NO_3_^-^ concentrations for the summer periods of 2000-2009 (N = 127 to 156, depending on the stream gage), which covered a range of hydrological conditions at each gage, but exhibited limited variation in average NO_3_^-^ concentrations. Because of the shorter period of record at the fourth gage, Dead Run at Franklintown, Maryland (USGS 01589330), we used average summer NO_3_^-^ concentration data in the Dead Run Watershed from Pennino et al. [[*Pennino et al.*, 2014](#_ENREF_10)]. We applied these NO_3_^-^ concentrations as boundary conditions for each of the 50 subbasins based upon values estimated at the stream gage within the closest downstream proximity.

WASP requires estimates of multiple hydraulic variables for each modeled stream segment. We estimated widths and depths for each stream segment in the sub-basins using standard regional regression equations from techniques used in the Grid Based Mercury Model (see below) [[*Dai et al.*, 2005](#_ENREF_5)]. We modified the width regression equation based upon measurements in the watershed during summer 2008 (S. Kaushal, unpublished data, 2013). We estimated slopes for each modeling segment by extracting elevation data at each segment’s upstream and downstream point using Spatial Analyst in ArcGIS 9.3.1 (ESRI, Redlands, California) and calculating the difference in distance (a constant 200 m) and elevation between the points. The full set of WASP stream segment hydraulic input parameters are listed in Table S1.

Our simulation approach required several assumptions and simplifications. First, WASP simulates varying depths for each stream segment using the continuity equation for the mass of water and based on roughness coefficients and stream widths. However, to best generalize measurements of uptake across the watershed, we did not simulate variations in uptake rates with depth. Other potentially important features of the system that were not modeled include hydrologic exchanges between the channel, floodplain, and subsurface waters [[*Helton et al.*, 2011](#_ENREF_6)].

2.3 Stream Burial Scenarios

We used the following stream burial scenarios to assess changes in NO_3_^-^ export at the watershed outlet: (1) Increasing the proportion of the stream network that was buried by 5% increments. Burial was evenly distributed across the 200 m stream segments; (2) Concentrated burial that reflects potential suburban development (all 85 segments in suburban subbasins (FS03); 1700 m buried, or 12.2% of the total stream network length), and (3) Concentrated burial that reflects urban development along the lower watershed mainstem (85 segments in urban subbasins (FS03) for a total of 1700 m of buried stream channel, same length used for the suburban scenario).

We also conducted two simulations to determine whether modeled changes in NO_3_^-^ export were a result of changes in N-removal rates due to burial, changes in hydrology resulting from burial, or a combination of the two. We held uptake rates constant and modified stream velocity via variations in the Manning’s roughness coefficient for the 50 subbasins in the first scenario and held hydrologic inputs constant and varied N-uptake rates in the second scenario. We based stream velocity and uptake rates upon whether the stream was buried or open.

2.4 WASP stream segment geomorphology calculations

Width (m) = 1.7^#^ * Drainage area (km^2^) ^0.3916##^ (3)

# = original coefficient was 2.933 (from Dai et al. [[*Dai et al.*, 2005](#_ENREF_5)]); final coefficient (1.7) was adjusted based on measurements from summer 2008 (S Kaushal, unpublished data)

## = estimated using techniques from Dai et al. [[*Dai et al.*, 2005](#_ENREF_5)]

Depth (m) = 0.33^#^ * Drainage area (km^2^) ^0.2964##^ (4)

## = estimated using techniques from Dai et al. [[*Dai et al.*, 2005](#_ENREF_5)]

**References**

Ambrose Jr, R. B. (1987), Modeling volatile organics in the Delaware Estuary, *J. Environ. Eng.*, *113*, 703-721.

Ambrose Jr, R. B. (1988), WASP4, A Hydrodynamic and WATER QUALITY MODEL—Model Theory, User’s Manual, and Programmer’s Guide. Technical Report. U.S. Environmental Protection Agency, Athens, GA. *Rep. EPA/600/3-87-039*.

Arnold, J. G., and P. M. Allen (1999), Automated methods for estimating baseflow and ground water recharge from streamflow records, *J. Am. Water Resour. As.*, *35*(2), 411-424.

Arnold, J. G., P. M. Allen, R. Muttiah, and G. Bernhardt (1995), Automated base flow separation and recession analysis techniques, *Ground Water*, *33*(6), 1010-1018.

Dai, T., R. B. Ambrose, K. Alvi, T. Wool, H. Manguerra, M. Choski, H. Yang, and S. Kraemer (2005), *Characterizing Spatial and Temporal Dynamics: Development of a Grid-Based Watershed Mercury Loading Model*, Moglen, Glenn E. (ed.), Managing Watersheds for Human and Natural Impacts: Engineering, Ecological, and Economic Challenges, Williamsburg, Virginia, 19-22 July 2005, ASCE Conference Proceedings, American Society of Civil Engineers, Reston, Virginia, doi:10.1061/40763(178)56.

Helton, A. M., et al. (2011), Thinking outside the channel: modeling nitrogen cycling in networked river ecosystems, *Front. Ecol. Environ.*, *9*(4), 229-238.

Johnston, J. M., et al. (2011), An integrated modeling framework for performing environmental assessments: Application to ecosystem services in the Albemarle-Pamlico basins (NC and VA, USA), *Ecol. Model.*, *222*(14), 2471-2484.

Knightes, C. D., E. M. Sunderland, M. C. Barber, J. M. Johnston, and R. B. Ambrose, Jr. (2009), Application of ecosystem-scale fate and bioaccumulation models to predict fish mercury response times to changes in atmospheric deposition, *Environ. Toxicol. Chem.*, *28*(4), 881-893.

Lin, Y., T. Larssen, R. D. Vogt, X. Feng, and H. Zhang (2011), Modelling transport and transformation of mercury fractions in heavily contaminated mountain streams by coupling a GIS-based hydrological model with a mercury chemistry model, *Sci. Total Environ.*, *409*(21), 4596-4605.

Pennino, M. J., S. S. Kaushal, J. J. Beaulieu, P. M. Mayer, and C. P. Arango (2014), Effects of urban stream burial on nitrogen uptake and ecosystem metabolism: implications for watershed nitrogen and carbon fluxes, *Biogeochemistry*, *121*(1), 247-269.
